# Facile Synthesis of Co_3_O_4_@CoO@Co Gradient Core@Shell Nanoparticles and Their Applications for Oxygen Evolution and Reduction in Alkaline Electrolytes

**DOI:** 10.3390/ma13122703

**Published:** 2020-06-13

**Authors:** Shih-Cheng Chou, Kuang-Chih Tso, Yi-Chieh Hsieh, Bo-Yao Sun, Jyh-Fu Lee, Pu-Wei Wu

**Affiliations:** 1 Department of Materials Science and Engineering, National Chiao Tung University, Hsinchu 300, Taiwan; chasers213@yahoo.com.tw (S.-C.C.); wchieh1219@gmail.com (Y.-C.H.); vul935552@gmail.com (B.-Y.S.); 2 Graduate Program of Accelerator Light Source, National Chiao Tung University, Hsihchu 300, Taiwan; scott22567@gmail.com; 3 National Synchrotron Radiation Research Center, Hsinchu 300, Taiwan; jflee@nsrrc.org.tw

**Keywords:** cobalt oxides, cobalt, core@shell nanoparticles, electrocatalyst, oxygen reduction reaction, oxygen evolution reaction, displacement reaction

## Abstract

We demonstrate a facile fabrication scheme for Co_3_O_4_@CoO@Co (gradient core@shell) nanoparticles on graphene and explore their electrocatalytic potentials for an oxygen evolution reaction (OER) and an oxygen reduction reaction (ORR) in alkaline electrolytes. The synthetic approach begins with the preparation of Co_3_O_4_ nanoparticles via a hydrothermal process, which is followed by a controlled hydrogen reduction treatment to render nanoparticles with radial constituents of Co_3_O_4_/CoO/Co (inside/outside). X-ray diffraction patterns confirm the formation of crystalline Co_3_O_4_ nanoparticles, and their gradual transformation to cubic CoO and fcc Co on the surface. Images from transmission electron microscope reveal a core@shell microstructure. These Co_3_O_4_@CoO@Co nanoparticles show impressive activities and durability for OER. For ORR electrocatalysis, the Co_3_O_4_@CoO@Co nanoparticles are subjected to a galvanic displacement reaction in which the surface Co atoms undergo oxidative dissolution for the reduction of Pt ions from the electrolyte to form Co_3_O_4_@Pt nanoparticles. With commercial Pt/C as a benchmark, we determine the ORR activities in sequence of Pt/C > Co_3_O_4_@Pt > Co_3_O_4_. Measurements from a rotation disk electrode at various rotation speeds indicate a 4-electron transfer path for Co_3_O_4_@Pt. In addition, the specific activity of Co_3_O_4_@Pt is more than two times greater than that of Pt/C.

## 1. Introduction

The development of electrocatalysts for oxygen electrochemistry has attracted considerable attention for many decades because both the oxygen evolution reaction (OER) and oxygen reduction reaction (ORR) play important roles in alternative energy sectors [1]. For example, during water electrolysis to produce pure hydrogen and oxygen, the OER is known to be the rate-determining step as considerable overpotential is often required. Therefore, it is of particular interest to develop effective electrocatalysts to minimize unnecessary energy loss. In fuel cells where the oxygen combines with hydrogen to produce electricity, the ORR is the most energy-consuming step and it reduces the efficiency of converting energy.

In research studies, the primary electrocatalysts for OER are conductive oxides such as RuO_2_, IrO_2_, and their composites [2,3]. It is because metallic electrocatalysts would suffer from oxidation during OER and their performances degrade steadily with time. Since both RuO_2_ and IrO_2_ are oxides of noble metals, there is a strong motivation to develop less-expensive alternatives for comparable OER activities [4,5,6,7,8]. For example, the cubic Co_3_O_4_ was reported to reveal impressive OER performance in alkaline electrolytes [9]. Another approach that has been widely explored is the fabrication of core-shell nanoparticles (core@shell) in which the inexpensive constituent occupies the core whereas the shell is covered by the element responsible for an electrochemical action [10,11]. This approach makes logical sense since the electrochemical reaction is an interfacial process where only the surface atoms matter. Moreover, it has been established that proper selection of a core constituent is able to enhance the electrocatalytic behavior of surface atoms via deliberate modification of the latter’s lattice parameter or energy band [12].

For ORR electrocatalysis, the Pt and Co have been studied for their chemical stability and impressive electrocatalytic activity in both acidic and alkaline electrolytes [13,14,15,16]. In the research, the Pt has been synthesized in a wide range of morphologies and nanostructures [17,18,19,20]. However, due to the expensive nature of Pt, it is rather critical to explore low-cost alternatives that could still deliver similar ORR performances. A straightforward solution is the formation of alloyed nanoparticles with binary (Pt-Ni, Pt-Fe, and Pt-Co) and tertiary (Pt-Fe-Co, Pt-Cu-Ni, and Pt-Fe-Ni) constituents [21,22,23,24,25,26]. On many occasions, these alloyed electrocatalysts not only reduce the Pt utilization but also demonstrate even stronger ORR activities [27,28,29,30]. An alternative route to reduce the Pt usage is the preparation of core@Pt nanoparticles since the heterogeneous core is able to alter both the electronic structure and the lattice parameter of surface Pt atoms, and, consequently, affects the ORR activities in distinct manners [31,32]. So far, the preparation of core@Pt nanoparticles is often achieved via a galvanic displacement reaction in which corrosive-prone atoms are pre-coated on selective cores, which was followed by their oxidative dissolution for reducing Pt ions from the electrolyte. For example, in our group, we adopted a Cu under-potential-deposition (UPD) to synthesize Pd_9_Ru@Cu nanoparticles and engaged the displacement reaction to fabricate Pd_9_Ru@Pt nanoparticles for ORR and methanol electro-oxidation activities in acidic electrolytes [33,34]. In the literature, the UPD process is a powerful tool to fabricate a core@shell microstructure by first depositing a Cu monolayer, which is followed by a displacement reaction in which the Cu is corrosively dissolved in conjunction with the reduction of selective ions from the electrolyte [35,36].

Previously, a wide variety of oxides such as rutiles, perovskites, and spinels have been investigated as the ORR electrocatalysts [37,38,39,40]. It is understood that the presence of cations with mixed valences in a spinel lattice could provide donor-acceptor chemisorption sites for reversible adsorption of oxygen. In addition, these mixed-valence oxides exhibit reasonable electric conductivity because of the relatively low activation energy required for electron hopping between neighboring cations. In the literature, the first documented report of oxide@Pt nanoparticles for ORR was demonstrated by Dhavale and Kurungot using Fe_2_O_3_ as the core [41]. Recently, the Co_3_O_4_ was reported to reveal impressive ORR activities with a similar four electron path to that of Pt [42]. Similar behaviors were reported in the spinel family of cobaltites (NiCo_2_O_4_ and MnCo_2_O_4_) [43,44]. Since the synthesis of Co_3_O_4_ nanoparticles is straightforward and its raw material cost is relatively inexpensive, it is possible to engineer the Co_3_O_4_ nanoparticles into gradient Co_3_O_4_@CoO@Co nanoparticles for direct OER electrocatalysis. Similar compositions have recently been demonstrated with impressive electrocatalytic activities [45,46]. In addition, instead of conventional Cu UPD, the surface-enriched Co atoms could be leveraged to initiate the displacement reaction for forming Co_3_O_4_@Pt nanoparticles for ORR actions.

In this work, we adopted the core@shell design to fabricate nanoparticles with radial composition of Co_3_O_4_/CoO/Co (inside/outside) and evaluated their OER activities in an alkaline electrolyte. These Co_3_O_4_@CoO@Co nanoparticles were produced by a controlled reduction of Co_3_O_4_ nanoparticles derived from hydrothermal synthesis. In addition, we employed the displacement reaction to synthesize Co_3_O_4_@Pt nanoparticles and evaluated their ORR activities. Material characterization and electrochemical analysis were conducted, and the results were thoroughly discussed.

## 2. Materials and Methods

### 2.1. Synthesis of Co_3_O_4_/CoO/Co and Co_3_O_4_@Pt Nanoparticles

The synthesis of Co_3_O_4_/CoO/Co nanoparticles started with the preparation of Co_3_O_4_ nanoparticles. First, 1.2 mL of 0.2 M Co(Ac)_2_ (98%, Alfa Aesar, Ward Hill, MA, USA), 0.5 mL of NH_4_OH (28%, J.T. Baker, Phillipsburg, PA, USA), 0.7 mL of deionized water, and 12 mg of graphene (Enerage Inc., Yilan, Taiwan) were mixed in 24 mL of ethanol at 25 °C. Subsequently, the mixture was kept at 80 °C under constant stirring for 10 h. Then, the mixture was transferred to a 40 mL autoclave for a hydrothermal process for 3 h at 150 °C. Afterward, the synthesized Co_3_O_4_ nanoparticles impregnated on the graphene (Co_3_O_4_/GN) were retrieved by vacuum filtration, which was followed by repeated rinsing in ethanol. Next, the Co_3_O_4_/GN underwent a mild reduction treatment (5%H_2_-95%Ar) at 350 °C for 15 min to reduce the surface Co_3_O_4_ to CoO and Co, which formed core@shell (Co_3_O_4_@CoO@Co) nanoparticles impregnated on the graphene (Co_3_O_4_@CoO@Co/GN).

To engage the galvanic displacement reaction between the Co_3_O_4_@CoO@Co/GN and Pt ions in the electrolyte, 5 mg of Co_3_O_4_@CoO@Co/GN was suspended in 5 mL of ethanol at 10 °C for 15 min, which was followed by the slow addition of 5 mL of 5 mM K_2_PtCl_4_ (98%, UniRegion Bio-Tech, Taipei, Taiwan) aqueous solution. The galvanic displacement reaction lasted for 30 min, which allowed the formation of Pt nanoparticles on the surface of Co_3_O_4_ nanoparticles. This formed Co_3_O_4_@Pt impregnated on the graphene (Co_3_O_4_@Pt/GN). Afterward, the resulting powders were collected by centrifugation and washed with excessive deionized water and ethanol.

### 2.2. Materials Characterization

X-ray diffraction (XRD, Bruker, Billerica, MA, USA) patterns were acquired by a Bruker D2 Phaser system equipped with a Cu Kα radiation source (λ = 1.54 Å) and the samples were prepared by dropping the ink slurry on a Si wafer. The resulting diffraction peaks were used to identify a relevant phase and particle size for Co_3_O_4_, Co_3_O_4_@CoO@Co, and Co_3_O_4_@Pt. A transmission electron microscope (TEM, FEI Tecnai F20, Philips/FEI, Amsterdam, Netherlands) was employed to observe the size and morphology of the electrocatalysts and the TEM samples were prepared by dropping the ink slurry onto Cu grids. X-ray absorption spectroscopy (XAS, NSRRC, Hsinchu, Taiwan) was adopted to determine the oxidation state of the Co before and after galvanic displacement reaction. The XAS experiments were performed at the BL17C beam line of the National Synchrotron Radiation Research Center (NSRRC) in Hsinchu, Taiwan, China. The standard operation conditions were 1.5 GeV and 300−360 mA. The XAS profiles were obtained in a fluorescence mode at 25 °C in air with Ni and Cr foil serving as fluorescence filter screening photons entering the fluorescence detector. The resulting X-ray absorption near-edge spectra (XANES) were processed by a program (Athena, version 0.8.056) in which the Co K-edge absorption steps were normalized to unify with the removal of background signals. Commercially available Co, CoO, and Co_3_O_4_ were adopted as the reference materials. Detailed XAS processing protocols were reported earlier [47].

### 2.3. Electrochemical Analysis 

To determine OER activities, 5 mg of Co_3_O_4_/GN or Co_3_O_4_@CoO@Co/GN, 0.016 mL of Nafion ionomers solution (5 wt %), and 1 mL of water/isopropanol (1:1 vol/vol) were mixed and sonicated for 1 h. Next, 0.005 mL of ink (25 µg electrocatalyst) was deposited on a rotating disk electrode (RDE, glassy carbon electrode with 5 mm in diameter) serving as the working electrode. A saturated calomel electrode (SCE) and a Pt foil (3 × 3 cm^2^) were used as the reference and counter electrode, respectively. The electrolyte was aqueous 0.1 M potassium hydroxide (KOH) aqueous solution and was saturated with oxygen for 30 min. The transient OER activities were determined by conducting linear *i*-V polarization between 0 and 1 V (vs. SCE) at 5 mV s^−1^. The potential for the reversible hydrogen electrode (RHE) was 0.99 V (vs. SCE). In our *i*-V profiles, the potentials were plotted against the RHE. Evaluation on the durability of Co_3_O_4_@CoO@Co/GN was conducted by recording the *i*-V polarization profile after 1000 cyclic voltammetric (CV) scans from 0.2 to 0.3 V (vs. RHE) at 50 mV s^−1^ in aqueous 0.1 M KOH solution.

For ORR activities, 5 mg of electrocatalyst (Co_3_O_4_/GN or Co_3_O_4_@Pt/GN), 0.016 mL of Nafion ionomers solution (5 wt %), and 1 mL of water/isopropanol (1:1 vol/vol) were mixed and sonicated for 1 h. On an RDE, 0.004 mL of ink (20 µg electrocatalyst) was deposited. The SCE was used as the reference electrode. The ORR experiments were carried out in a three-electrode cell using a Pt foil (3 × 3 cm^2^) as the counter electrode and 0.1 M KOH aqueous solution as the electrolyte. Both working and counter electrodes were in the same compartment. Before the experiments, the electrolyte was saturated with oxygen for 30 min. During the measurements, the oxygen was still flowing. The ORR activities for both Co_3_O_4_/GN and Co_3_O_4_@Pt/GN were obtained by conducting linear *i*-V polarization between 0.11 and −0.99 V (vs SCE) at a scan rate of 5 mV s^−1^. The electrochemical active surface area (ECSA) of Co_3_O_4_@Pt/GN was determined by imposing cyclic voltammetric (CV) scans between 0 and 1.5 V (vs. RHE) at 50 mV s^−1^ in a de-aerated 0.1 M KOH aqueous solution. The ECSA was determined by dividing the coulomb charge associated with the hydrogen UPD region between 0.05 and 0.4 V (vs. RHE) by 210 µC cm^−2^_Pt_. All the electrochemical experiments were conducted by a potentiostat (VersaSTAT4) at 25 °C. For both ORR activities and ECSA, commercially available Pt/C (20 wt % Pt on XC72R, BASF SE, Ludwigshafen, Germany) was also analyzed for a comparison purpose.

## 3. Results

### 3.1. Characterizations of Co_3_O_4_@CoO@Co for OER Activities

**Figure 1:**
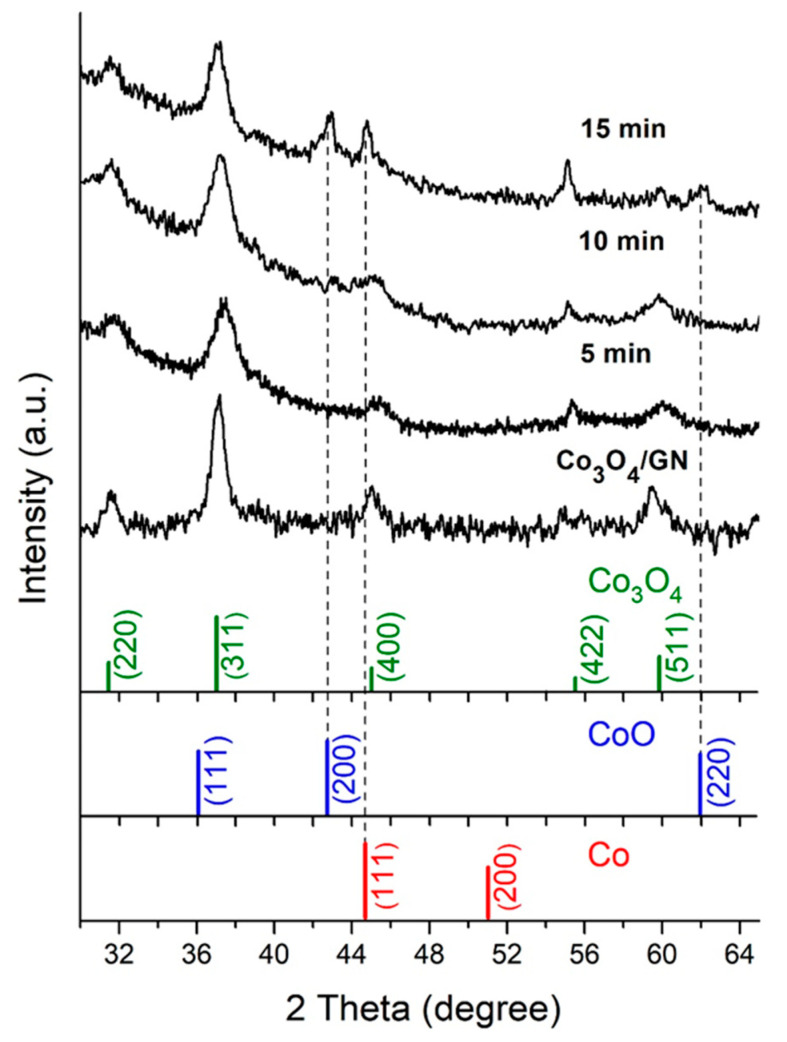
The XRD patterns of as-synthesized Co_3_O_4_ nanoparticles and after hydrogen reduction treatment at 350 °C in 5%H_2_-95%Ar for 5, 10, and 15 min, respectively. The standard materials of cubic Co_3_O_4_ (JCPDS: 073–1701), fcc Co (JCPDS: 89–7093), and cubic CoO (JCPDS: 70–2855) are also shown.

Figure 1 displays the XRD patterns for Co_3_O_4_ before and after the reduction treatment (5, 10, and 15 min) as well as standard Co, CoO, and Co_3_O_4_ for a comparison purpose. Apparently, the as-synthesized Co_3_O_4_ exhibited diffraction peaks and intensities that were consistent with those of standard cubic Co_3_O_4_ (Joint Committee on Powder Diffraction Standard, JCPDS: 073–1701), suggesting its isotropic nature. For samples after hydrogen treatment, the predominant peaks were still attributed to those of Co_3_O_4_, but additional diffraction signals were observed for samples after hydrogen treatments of 10 and 15 min, and they were associated with CoO and Co, respectively. For example, for the sample of a 15-min reduction treatment, the strongest peak of CoO was recorded at 2θ of 42.5° for the (200) plane whereas the strongest peak of Co was also observed at 2θ of 44.3° for the (111) plane. It is noted that. for both CoO and Co, only their respective strongest peaks were observed, which suggested their absolute amounts were relatively insignificant as compared to that of Co_3_O_4_. This suited our purpose as the Co_3_O_4_ core was nicely maintained without experiencing excessive reduction. We were able to estimate the grain size of Co_3_O_4_, CoO, and Co using Scherrer’s equation on their respective strongest diffraction peaks, and determined their size was 9.3 nm (Co_3_O_4_), 2.8 nm (CoO), and 4.1 nm (Co), respectively. The presence of surface Co atoms was further confirmed by a simple magnetic test in which the dried Co_3_O_4_@CoO@Co powders were easily attracted toward a magnet. Our results indicated that the reduction treatment of 15 min was successful in reducing the surface of Co_3_O_4_ nanoparticles to CoO and Co. Considering the reduction reaction was driven by the hydrogen molecules, which diffused inward, it is reasonable to expect the radial composition of our sample to be Co_3_O_4_/CoO/Co (inside-outside). Because the samples after 5-min and 10-min reduction treatments did not exhibit convincing evidence for the formation of CoO and Co. The sample of a 15-min reduction treatment was selected for subsequent characterization and electrochemical analysis. However, it is noted that further studies are warranted to conduct extended heat treatments. Therefore, the amount of CoO and Co could be varied and their effects for OER could be elucidated.

**Figure 2:**
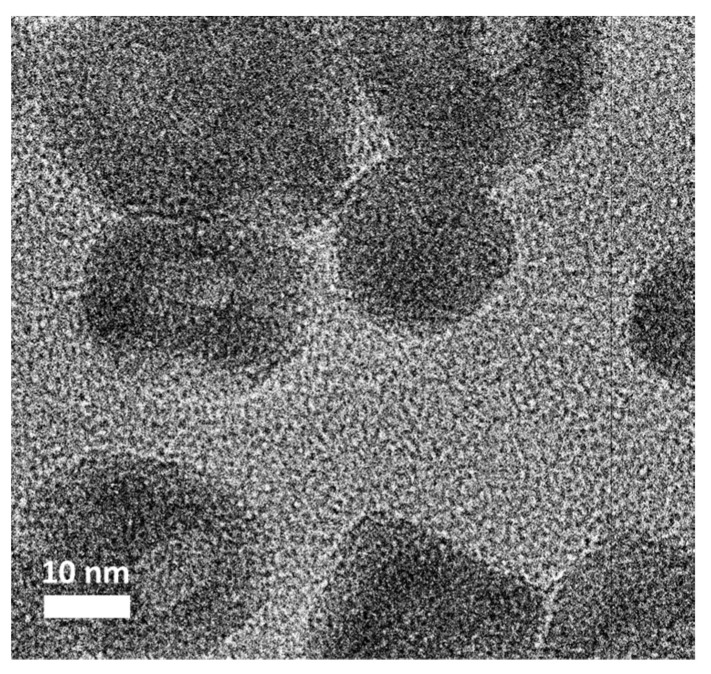
The TEM image of Co_3_O_4_@CoO@Co/GN.

Figure 2 displays the TEM image of Co_3_O_4_@CoO@Co/GN. Apparently, the Co_3_O_4_@CoO@Co nanoparticles exhibited a nearly spherical shape with a size of 24.3 ± 4.8 nm. In typical TEM imaging, areas with a darker appearance represent structures with higher mass density. In our sample, the metallic Co on the shell was expected to have a greater mass density, and, thus, revealed a darker image as compared to that of Co_3_O_4_ at the core, which was smaller in mass density, and, thus, appeared as a lighter image. In between, there was the formation of CoO whose image became indistinguishable with that of Co_3_O_4_ because of similar mass density. A closer look at this TEM image indicated that the average diameter for the Co_3_O_4_ core was 10.4 ± 3.8 nm and the Co shell thickness was 7.7 ± 2.5 nm. We did not perform compositional analysis on X-ray photoelectron spectroscopy because the Co_3_O_4_@CoO@Co revealed a strong magnetic behavior that precluded its possible diagnosis.

**Figure 3:**
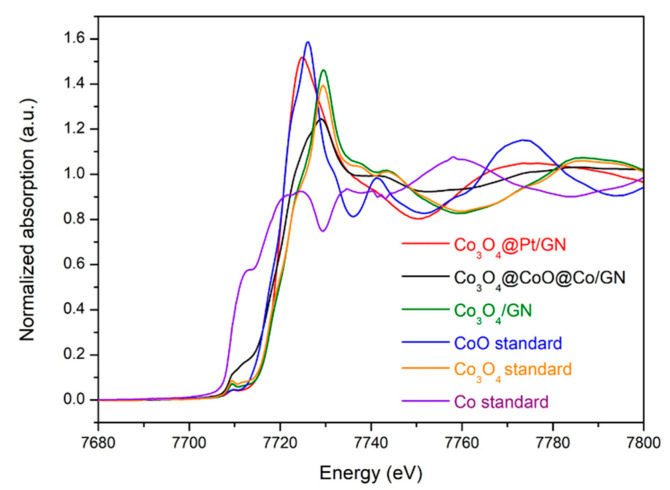
The Co K-edge X-ray absorption near-edge spectra (XANES) profiles for Co_3_O_4_/GN, Co_3_O_4_@CoO@Co/GN, and Co_3_O_4_@Pt/GN, as well as standard materials of Co, CoO, and Co_3_O_4_.

The XAS is a powerful tool in providing qualitative information about the average oxidation state and local atomic structure of the absorbing atoms. Therefore, the XAS was utilized to investigate the absorbance of Co in Co_3_O_4_/GN, and Co_3_O_4_@CoO@Co/GN, and the resulting Co XANES spectra are displayed in Figure 3, along with standard materials of Co, CoO, and Co_3_O_4_ for comparison purposes. In general, for K-edge XANES, the absolute magnitude of the absorption energy is proportional to the oxidation state of the absorbing atom. For example, Co with a higher oxidation state reveals a greater absorption energy. Therefore, these XANES profiles were used to compare with those of standard materials to determine the oxidation state of Co qualitatively. The oxidation state of Co in Co, CoO, and Co_3_O_4_ is 0, +2, and +2.67, respectively. The K-edge absorption energy of standard materials appeared in order of Co_3_O_4_ > CoO > Co. For the Co_3_O_4_/GN sample, its XANES profile was identical to that of standard Co_3_O_4_. After the reduction treatment, the XANES profile for Co_3_O_4_@CoO@Co/GN was shifted slightly to lower energy, becoming similar to that of the CoO standard. This result was anticipated as the XAS recorded the average oxidation state of Co.

**Figure 4:**
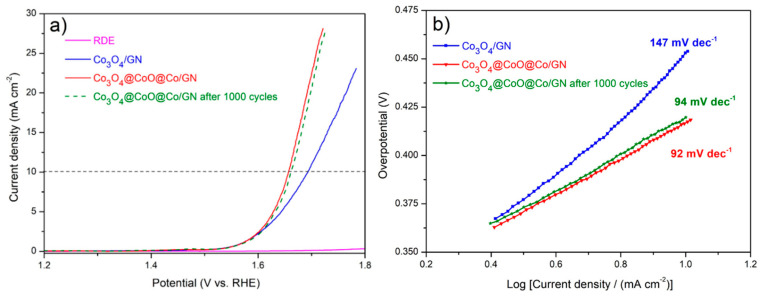
(**a**) The initial oxygen revolution reaction (OER) *i*-V curves for rotating disc electrode (RDE), Co_3_O_4_/GN, and Co_3_O_4_@CoO@Co/GN (before and after 1000 cyclic voltammetric (CV) cycles). (**b**) The Tafel plots for Co_3_O_4_/GN and Co_3_O_4_@CoO@Co/GN (before and after 1000 CV cycles).

Figure 4a displays the OER *i*-V profiles for RDE and Co_3_O_4_/GN as well as Co_3_O_4_@CoO@Co/GN before and after 1000 CV cycles. As shown, the RDE exhibited negligible OER activity because the glassy carbon is not electrocatalytically active. At 10 mA cm^−2^, the Co_3_O_4_@CoO@Co/GN revealed a smaller overpotential as compared to that of Co_3_O_4_. This result was consistent with what was reported by Hang et al. in which metallic Co was used to improve the electrical conductivity of Co_3_O_4_ for improved OER activities [48]. In addition, the Co_3_O_4_@CoO@Co/GN showed impressive durability with similar *i*-V profiles after 1000 cycles. It is noted that our testing methods are consistent with what was used in the literature [49]. Therefore, our results were reliable and reproducible. To further explore their electrocatalytic behavior, these *i*-V profiles are replotted in the Tafel curve, as shown in Figure 4b. The Co_3_O_4_@CoO@Co/GN demonstrated a Tafel slope of 92 mV dec^−1^, which was relatively lower than that of Co_3_O_4_/GN at 147 mV dec^−1^. This Tafel slope was similar to what was reported earlier [48]. The determination of exact electrocatalytic contributions from CoO and Co in OER is not feasible because the Co atoms experience complicated oxidation and reduction steps during OER actions. In short, we demonstrated a simple approach to fabricate gradient nanoparticles under deliberate hydrogen reduction, and the gradient nanoparticles revealed effective improvement in OER relative to the untreated oxide nanoparticles.

### 3.2. Characterization of Co_3_O_4_@Pt/GN for ORR Activities

**Figure 5:**
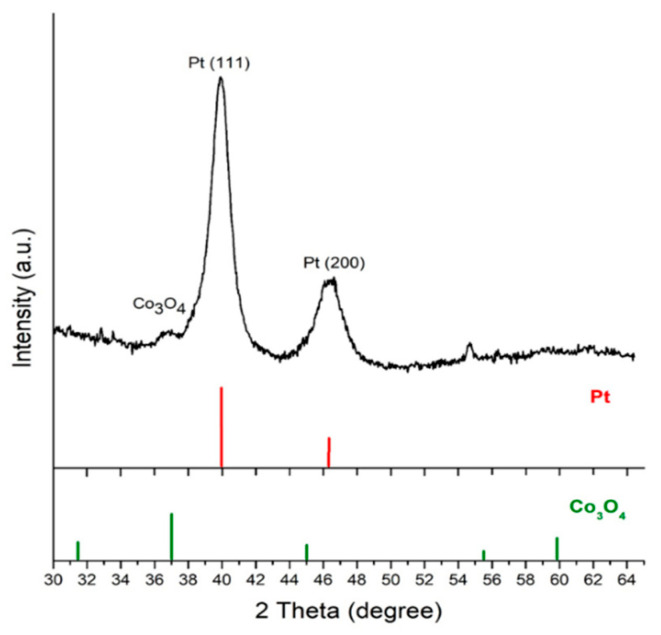
The XRD pattern of Co_3_O_4_@Pt/GN as well as JCPDS of cubic Co_3_O_4_ (JCPDS: 073–1701) and fcc Pt (JCPDS: 1–87–636).

The synthesis of Co_3_O_4_@Pt nanoparticles was achieved by a deliberate displacement reaction between Co and Pt ions, which proceeded via Co + [PtCl_4_]^2−^ → Pt + Co^2+^ + 4Cl^−^. The difference between the E^0^ value of Pt/[PtCl_4_]^2−^ (0.758 V vs. Normal hydrogen electrode, NHE) and Co/Co^2+^ (−0.28 V vs. NHE) is sufficient for the displacement reaction to activate. The aqueous K_2_PtCl_4_ solution was in light yellow. After we finished the displacement reaction, the solution remained light yellow because excessive Pt ions were used in the electrolyte for the displacement reaction. The presence of Pt on the Co_3_O_4_@Pt/GN was verified by XRD. The resulting diffraction pattern along with those of standard fcc Pt (JCPDS: 1–87–636) and cubic Co_3_O_4_ (JCPDS: 073–1701) are displayed in Figure 5. This diffraction pattern was plotted between 2θ of 30–65° to minimize unnecessary interference from graphene at 2θ of 27°. As shown, the predominant peaks at 2θ of 40.5° and 47° were associated with Pt (111) and Pt (200), respectively, and their relative intensity agreed well with those of standard Pt. Unexpectedly, the strongest peak for (311) Co_3_O_4_, located at 2θ of 37.2°, was rather weak, and the CoO signal was clearly absent. We realized that CoO was dissolved during the displacement reaction because it was chemically unstable in an acidic solution. Despite the diffraction ability of metallic Pt being much larger than that of Co_3_O_4_, judging from the large intensity contrast between the (311) Co_3_O_4_ and (111) Pt, we surmised that the amount of Pt on the Co_3_O_4_@Pt/GN was rather substantial. In addition, the estimation of the Pt crystallite size from Sherrer’s equation using the Pt (111) plane was 6.2 nm.

**Figure 6:**
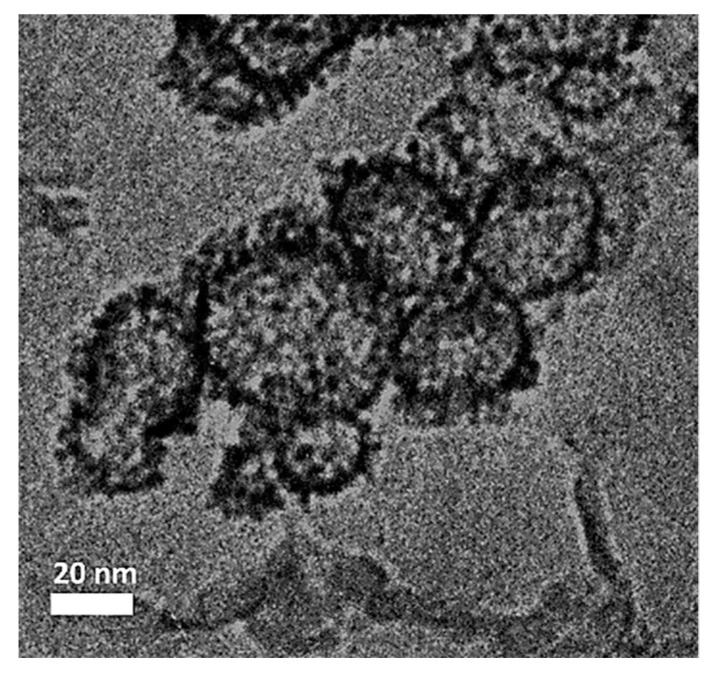
The TEM images of Co_3_O_4_@Pt/GN.

Figure 6 exhibits the TEM image for Co_3_O_4_@Pt/GN. As expected, the Co_3_O_4_ at the core revealed a lighter image whereas the Pt occupied the outer shell in a darker image. Notably, the Pt shell appeared as aggregates of Pt nanoparticles and the primary Pt nanoparticle size was 8.3 ± 1.2 nm. This value was in reasonable agreement with that of XRD analysis in Figure 5. In addition, the aggregates of Pt nanoparticles did not fully encapsulate the Co_3_O_4_ core and, therefore, partial exposure of Co_3_O_4_ to the electrolyte was possible.

Successful formation of Co_3_O_4_@Pt nanoparticles could be validated by their XAS profile. As shown in Figure 3, after the completion of the displacement reaction, the XANES profile for Co_3_O_4_@Pt/GN demonstrated a higher oxidation state than that of Co_3_O_4_@CoO@Co/GN. This phenomenon was anticipated because the metallic cobalt and CoO in Co_3_O_4_@CoO@Co was replaced by Pt, which led to the increase of the average oxidation state of the remaining Co atoms. The Pt had its characteristic K-edge absorption energy at 78,393 eV and L_III_-edge absorption energy at 11,564 eV. These peaks were not shown in this Co K-edge XANES profile.

**Figure 7:**
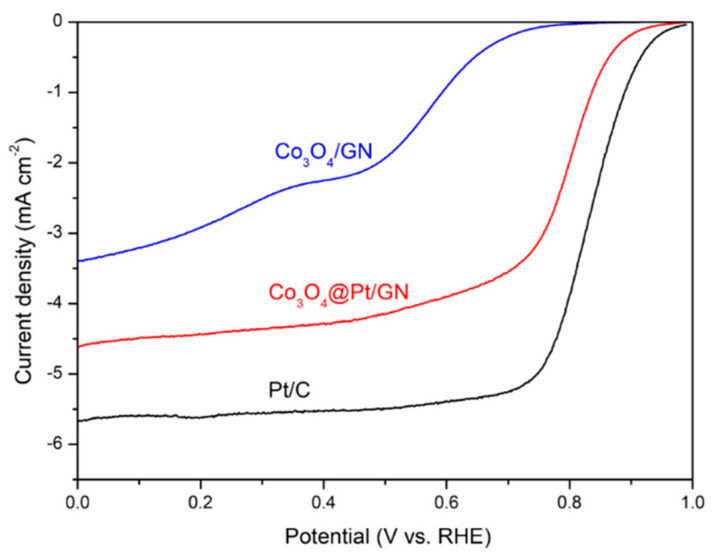
The oxygen reduction reaction (ORR) activities in apparent current density for Co_3_O_4_/graphene (GN), Co_3_O_4_@Pt/GN, and Pt/C. The experiments were conducted in an oxygen-saturated 0.1 M KOH aqueous solution at 1600 rpm and a scan rate of 5 mV s^−1^.

The ORR activities in apparent current density from Co_3_O_4_/GN and Co_3_O_4_@Pt/GN are shown in Figure 7 along with commercially available Pt/C for a comparison purpose. The apparent current density is based on the actual geometric area of RDE. According to the research, at a potential below 0.6 V, the ORR curve is under mass transport control limited by the diffusion of dissolved oxygen in the electrolyte, whereas, at a potential between 0.8 and 1 V, the ORR response is controlled by the kinetics of electrocatalysts [50]. Therefore, the most common method to compare the ORR activities of electrocatalysts is based on the half-way potential, which is defined by the potential at the half magnitude of limiting current. Generally, a larger half-way potential indicates a greater ORR activity. For Pt/C, at a rotation speed of 1600 rpm, the diffusion-limiting current was approaching 5.6 mA cm^−2^, which is a value that was close to what was reported for Pt previously [51,52]. For Co_3_O_4_/GN, the current response appeared in two distinct stages, which indicated that two separate steps were involved in the ORR because of a weak electrocatalytic activity [42]. However, the Co_3_O_4_@Pt/GN revealed a similar current response like that of Pt, albeit with a smaller limiting current. Nevertheless, the Co_3_O_4_@Pt/GN behaved much better than that of Co_3_O_4_/GN, which showed the advantage of depositing Pt as the shell layer. From Figure 7, the half-way potential for Co_3_O_4_/GN, Co_3_O_4_@Pt/GN, and Pt/C was 0.53, 0.79, and 0.83 V, respectively. The minor difference between Co_3_O_4_@Pt/GN and Pt/C was due to the incomplete coverage of Pt on the Co_3_O_4_. It is noted that, in our study, the GN was employed as the support for Co_3_O_4_@CoO@Co nanoparticles to reside on. In electrocatalysis, electrocatalysts in nanoparticulate forms are often impregnated on carbonaceous materials for better distribution and utilization. In the literature, the GN has been used as the substrate to support electrocatalysts for many electrochemical reactions [53,54].

To further investigate the kinetics of Co_3_O_4_@Pt/GN, we employed the Koutecky-Levich equation, which is listed below, to calculate the electron transfer number.

1/*i* = 1/*i_kinetic_* + 1/*i_diffusion limit_* = 1/*i_kinetic_* + 1/0.62*nFAD_O2_*^2/3^*ω*^1/2^*v*^−1/6^*C_O2_* (1)

where the *i* is the experimentally-measured apparent current, the *i_diffusion limit_* is the diffusion limiting current due to the limitation of mass transport of dissolved oxygen in the 0.1 M aqueous KOH solution, the *i_kinetic_* is the kinetic current associated with the ORR activity, *n* is the number of electrons transferred in the ORR process, *F* is the Faraday constant, *A* is the reaction area of the RDE (0.196 cm^2^), *D_O2_* is the diffusivity of dissolved oxygen in the 0.1 M aqueous KOH solution (1.9 × 10^−5^ cm^2^ s^−1^), *ω* is the rotation speed of the RDE, *v* is the kinematic viscosity of the 0.1 M aqueous KOH solution (1.09 × 10^−2^ cm^2^ s^−1^), and *C_O2_* is the concentration of dissolved oxygen in the 0.1 M aqueous KOH solution (1.2 × 10^−6^ mol cm^−3^).

**Figure 8:**
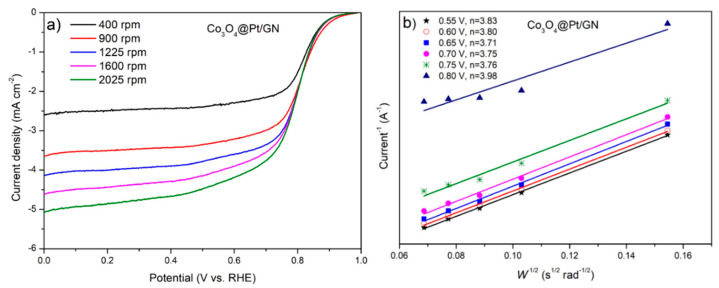
The rotating disc electrode (RDE) measurements for Co_3_O_4_@Pt/GN. (**a**) Oxygen reduction reaction (ORR) activity at various rotation speeds and (**b**) the Koutecky-Levich plot at different potentials.

Figure 8a exhibits the ORR curves of Co_3_O_4_@Pt/GN at various rotation speeds of RDE. Among these curves, the ORR curves at a voltage below 0.4 V were almost stabilized at the limiting currents whose magnitudes were proportional to the rotation speed. Figure 8b demonstrates the Koutecky-Levich plots at different potentials. These curves displayed a consistent pattern with an average slope of 12.2 (1/(mA × s^1/2^)). Accordingly, the electron transfer number, *n*, was close 3.8, which is a value rather close to 4 of typical Pt [52]. Our methodology was consistent with what was reported in the literature [42,55]. According to a prior study, the Co_3_O_4_ catalyzed the ORR in a 2.9 to 3.4-electron route [56]. Therefore, our results agreed well with the microstructural information from XRD and TEM that the surface of Co_3_O_4_@Pt/GN was predominantly Pt with a minor presence of Co_3_O_4_.

**Figure 9:**
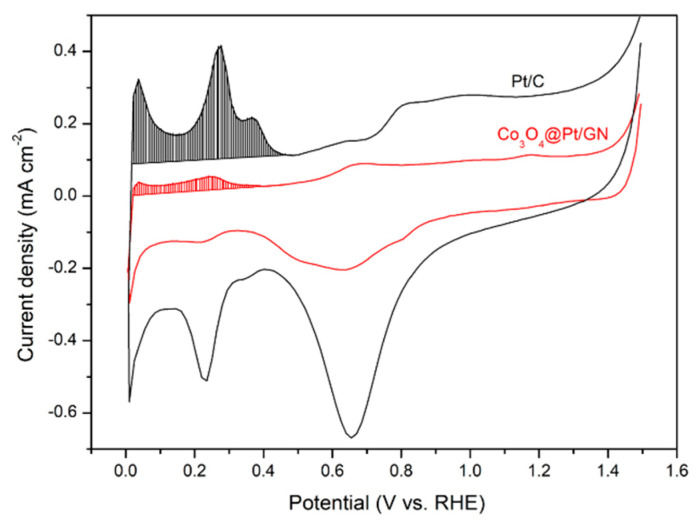
The cyclic voltammetric (CV) profiles for electrochemical active surface area (ECSA) measurements of Co_3_O_4_@Pt/GN and Pt/C.

Figure 9 demonstrates the CV curves for ECSA measurements in which the highlighted area was used to estimate the ECSA of Co_3_O_4_@Pt/GN and Pt/C, respectively. Between them, the Pt/C exhibited a significantly larger CV profile in both capacitive response of the carbon substrate and the redox behavior of Pt. The resulting ECSA for Co_3_O_4_@Pt/GN and Pt/C was 1.11 and 8.96 cm^2^, respectively. This smaller ECSA for our Co_3_O_4_@Pt/GN was expected as the Pt nanoparticles were aggregated on the Co_3_O_4_ surface whereas, in commercial Pt/C, the Pt nanoparticles were homogeneously distributed on a carbonaceous substrate.

Since the electrochemical reaction is an interfacial phenomenon, the magnitude of ECSA becomes an important factor as a greater ECSA typically leads to a larger electrocatalytic current. Therefore, to compare different electrocatalysts fairly, it is necessary to obtain the specific activity, which includes a parameter defined as *I_k_/ECSA*, where the *I_k_* is the kinetic current. In our case, the specific activity of Co_3_O_4_@Pt/GN and Pt/C was 0.549 and 0.248 mA cm^−2^_Pt_. In the literature, the rationale behind the enhanced electrocatalytic activity of core@Pt nanoparticles involves both electronic and structural effects. The electronic effect is concerned with the difference in the electronegativity between the core and Pt that shifts the d-band center and, thus, alters the electrocatalytic activity. The structural effect entails the changeup in the lattice constant of Pt because the core constituent adopts a different crystal structure. In the case of alloyed Pt nanoparticles, there is a mechanism known as a ligand effect in which the Pt and its alloying constituent cooperate in completing the electrocatalytic action. In our case, from the TEM and XRD analysis, we surmised that both electronic and structural effects were negligible.

In alkaline electrolytes, the magnitude of ORR activities for Pt, Co_3_O_4_, and GN is in the order of Pt > Co_3_O_4_ > GN. In our case, we prepared the core@shell structure in Co_3_O_4_@Pt so the Pt nanoparticles were anticipated to be used more effectively. The Co_3_O_4_ was adopted as a chemically stable core that functioned as a catalyst support but was more electrocatalytically active than that of GN. In practical fuel cell devices, during long-term operation, the detachment/poisoning of Pt nanoparticles become inevitable. Therefore, the Co_3_O_4_ is expected to provide moderate ORR activities at a relatively low cost to relieve the undesirable degradation in fuel cell performances. In short, we explored a simple mild reduction treatment instead of conventional Cu UPD to produce Co_3_O_4_@Pt nanoparticles for ORR action.

## 4. Conclusions

We successfully fabricated Co_3_O_4_@CoO@Co nanoparticles and evaluated their OER activities in an alkaline electrolyte. The Co_3_O_4_ nanoparticles were synthesized by a hydrothermal process, which was followed by a mild hydrogen reduction to produce Co_3_O_4_@CoO@Co nanoparticles. The XRD patterns confirmed the presence of crystalline Co_3_O_4_, CoO, and Co, and the TEM image confirmed a core-shell structure. In OER *i*-V profiles, the Co_3_O_4_@CoO@Co/GN revealed an impressive transient behavior and durability with a Tafel slope near 92 mV dec^−1^. The Co_3_O_4_@CoO@Co nanoparticles were successfully converted to Co_3_O_4_@Pt nanoparticles via a displacement reaction. The TEM image showed that the Pt existed as nanoparticles aggregated on the surface of Co_3_O_4_ with a coverage ratio of 90%. The ORR activity followed the sequence of Pt/C > Co_3_O_4_@Pt/GN > Co_3_O_4_/GN. Measurements from the RDE at various rotation speeds indicated a 4-electron transfer path for Co_3_O_4_@Pt/GN. The specific activity of Co_3_O_4_@Pt/GN was more than two times greater than that of Pt/C.
